# Psychometric properties of a short version of Lee Fatigue Scale used as a generic PROM in persons with stroke or osteoarthritis: assessment using a Rasch analysis approach

**DOI:** 10.1186/s12955-020-01419-8

**Published:** 2020-06-05

**Authors:** Line Kildal Bragstad, Anners Lerdal, Caryl L. Gay, Marit Kirkevold, Kathryn A. Lee, Maren Falch Lindberg, Ingrid Johansen Skogestad, Ellen Gabrielsen Hjelle, Unni Sveen, Anders Kottorp

**Affiliations:** 1 Department of Geriatric Medicine, Oslo University Hospital, P.O Box 4956, Ullevaal, Nydalen, 0424 Oslo, Norway; 2 Department of Nursing Science and Research Center for Habilitation and Rehabilitation Services and Models (CHARM), University of Oslo, Faculty of Medicine, Institute of Health and Society, P.O. Box 1130, Blindern, N-0318 Oslo, Norway; 3 Department of Nursing Science, University of Oslo, Faculty of Medicine, Institute of Health and Society, P.O. Box. 1130, Blindern, N-0318 Oslo, Norway; 4 Department for Patient Safety and Research, Lovisenberg Diaconal Hospital, P.O. Box 04970, Nydalen, N-0440 Oslo, Norway; 5 Department of Family Health Care Nursing, University of California, San Francisco, 2 Koret Way, San Francisco, CA 94143 USA; 6 Department for Surgery, Lovisenberg Diaconal Hospital, P.O. Box 04970, Nydalen, N-0440 Oslo, Norway; 7 Department for Medicine, Lovisenberg Diaconal Hospital, P.O. Box 04970, Nydalen, N-0440 Oslo, Norway; 8 Faculty of Health Sciences, Oslo Metropolitan University, P.O. Box 4, St. Olavs Plass, N-0130 Oslo, Norway; 9 Department of Physical Medicine and Rehabilitation, Oslo University Hospital, P.O. Box 4956, Nydalen, N-0424 Oslo, Norway; 10 Faculty of Health and Society, Malmö University, 205 06 Malmö, Sweden

**Keywords:** Fatigue, Psychometrics, Rasch analysis, Measurement, Stroke, Osteoarthritis, Total knee arthroplasty, Health-related quality of life

## Abstract

**Background:**

Fatigue is a common symptom associated with a wide range of diseases and needs to be more thoroughly studied*.* To minimise patient burden and to enhance response rates in research studies, patient-reported outcome measures (PROM) need to be as short as possible, without sacrificing reliability and validity. It is also important to have a generic measure that can be used for comparisons across different patient populations. Thus, the aim of this secondary analysis was to evaluate the psychometric properties of the Norwegian 5-item version of the Lee Fatigue Scale (LFS) in two distinct patient populations.

**Methods:**

The sample was obtained from two different Norwegian studies and included patients 4–6 weeks after stroke (*n* = 322) and patients with osteoarthritis on a waiting list for total knee arthroplasty (*n* = 203). Fatigue severity was rated by five items from the Norwegian version of the LFS, rating each item on a numeric rating scale from 1 to 10. Rasch analysis was used to evaluate the psychometric properties of the 5-item scale across the two patient samples.

**Results:**

Three of the five LFS items (“tired”, “fatigued” and “worn out”) showed acceptable internal scale validity as they met the set criterion for goodness-of-fit after removal of two items with unacceptable goodness-of-fit to the Rasch model. The 3-item LFS explained 81.6% of the variance, demonstrated acceptable unidimensionality, could separate the fatigue responses into three distinct severity groups and had no differential functioning with regard to disease group. The 3-item version of the LFS had a higher separation index and better internal consistency reliability than the 5-item version.

**Conclusions:**

A 3-item version of the LFS demonstrated acceptable psychometric properties in two distinct samples of patients, suggesting it may be useful as a brief generic measure of fatigue severity.

**Trial registration:**

Clinicaltrials.gov: NCT02338869; registered 10/04/2014 (stroke study).

## Background

Fatigue is a common symptom associated with a wide range of chronic diseases [1] and has been frequently studied in many different patient populations. Fatigue has been defined as a sense of exhaustion, lack of energy, or tiredness distinct from sleepiness, sadness, or weakness [2, 3]. To minimise patient burden and to enhance response rates in research studies, a short, yet valid and reliable patient-reported outcome measure (PROM) for fatigue is important for the feasibility of studies on fatigue. Further, in order to compare health status across medical conditions, we need to know whether a PROM for fatigue can be used as a generic measure in populations as diverse as patients affected by stroke and patients living with the consequences of osteoarthritis.

One of the frequently used PROMs for fatigue is the 13-item Lee Fatigue Scale (LFS). It has primarily been used among adults with cancer [4, 5] and human immunodeficiency virus (HIV) [6, 7]. However, it has also been used in other populations such as patients admitted to intensive care units [8], undergoing knee arthroplasty [9], stroke [10] and living with chronic obstructive pulmonary disease [11]. Nonetheless, PROMs with strong psychometric properties in one population need to be evaluated for use in other patient populations. We have previously shown how the psychometric properties of the LFS can vary in different populations and between countries [12], but these types of studies are rarely published. Rather, PROMs are often evaluated psychometrically in a single patient population, and then applied to other populations without further psychometric testing. Moreover, only PROMs that demonstrate strong and stable psychometric properties across a broad range of diverse patient populations should be considered a generic measure suitable for use in any patient population.

We have previously evaluated the English version of the LFS in samples of patients with cancer [13] and people with HIV [14]. Using Rasch analysis, we also reduced the full version of the LFS (13 items) to a short version (5 items) with satisfactory validity and reliability [2]. That study showed that the short version yielded similar fatigue severity ratings as the full scale for 95% of patients, had sufficient sensitivity to separate the responses into three distinct fatigue groups (low, moderate and high severity), and demonstrated unidimensionality and internal scale validity [2]. In research studies and in clinical practice, short instruments are generally preferred in order to minimize the burden on participants and maximize adherence to the protocol.

In order to evaluate whether the LFS is suitable for use as a generic measure of fatigue severity, there is a need for further exploration of the psychometric properties of the LFS. The aim of this study was to evaluate the psychometric properties of the Norwegian 5-item version of the LFS in two different patient populations, adults with stroke and adults with osteoarthritis.

## Methods

### Design

This study has a cross-sectional design and includes initial pre-intervention data from two longitudinal studies, a multicentre randomised controlled trial evaluating the effect of an intervention promoting psychosocial well-being following stroke [10] and a longitudinal study investigating pain and other symptoms in patients with osteoarthritis undergoing total knee arthroplasty [15].

### Participants and setting

#### Stroke sample

A total of 322 adult stroke survivors recruited from 11 acute stroke or rehabilitation units in university hospitals and other local hospitals providing acute care in Norway were consented into the trial [10]. The inclusion criteria were: adults ≥ 18 years of age, acute stroke within 4 weeks prior to inclusion, medically stable, sufficient cognitive functioning to participate (assessed by their physician/stroke team), able to understand and speak Norwegian, and able to give informed consent. Exclusion criteria were moderate to severe dementia, and serious somatic or psychiatric disease, as these conditions would likely have interfered with the ability to provide informed consent or fully participate in the study protocol.

Initial pre-intervention data were collected in structured in-person assessment interviews within 6 weeks after stroke onset. The data collector recorded the participant’s responses consisting of demographics and 5 items from the LFS [16], in addition to other PROMs included in the trial [10]. Psychometric properties of the LFS at baseline will be reported in this study.

#### Osteoarthritis sample

A total of 203 patients with osteoarthritis who were admitted for total knee arthroplasty at a surgical clinic in Oslo, Norway were included in the study. The inclusion criteria were: adults ≥ 18 years of age, ability to read, write and understand Norwegian, and scheduled for unilateral primary total knee arthroplasty. Patients undergoing unicompartmental or revision surgery were excluded. A comprehensive description of the study participants and data collection measures has been reported elsewhere [15].

The initial data collection was performed prior to surgery, after admission to the hospital. Patients independently completed paper questionnaires assessing demographic characteristics, 5 items from the original LFS and several other measures included in the osteoarthritis study [15]. Psychometric properties of the LFS at baseline will be reported in this study.

### Lee fatigue scale

Fatigue severity was measured in both samples using the same 5-item LFS. The following items with the anchor phrases from the original 13-item version [16] were used: item 1 “*not at all tired*” to “*extremely tired*”, item 4 “*not at all fatigued*” to “*extremely fatigued*”, item 5 “*not at all worn out*” to “*extremely worn out*”, item 16 “*carry on a conversation is no effort at all*” to “*carrying on a conversation is a tremendous chore*”, and item 17 “*I have absolutely no desire to close my eyes*” to “*I have a tremendous desire to close my eyes*”. All items were rated on a numeric rating scale from 0 to 10; higher scores indicate higher fatigue severity. Items 1, 4 and 5 were also included in a short version of the LFS evaluated through Rasch analysis among women living with HIV [2]. Although items 16 and 17 were not included in that previous short form, they were found to support the scale’s unidemensionality and internal scale validity when the original 13-item version of the LFS was assessed among women with HIV [14].

### Statistical analysis

The analysis of the LFS was guided by the use of a Rasch rating scale model [17]. The Rasch model is a confirmatory model where the data has to meet the model requirement to form a valid and unidimensional measurement scale, as compared to other item response theory (IRT) models that are exploratory models aiming to describe the variance in the data. Due to a technical error in the scoring of the LFS in the stroke sample, a score of 0 or 1 was scored as 1. To obtain a similar rating scale for both samples, scores of 0 in the osteoarthritis sample were recoded as 1 so that both samples were scored on a rating scale of 1–10. Thus, the original rating scale of 0–10 was transformed to a 10-level rating scale for both samples. This rating scale has been used successfully in earlier Rasch analyses of the LFS [14]. The transformed 10 category raw scores from the 5-item LFS were analyzed using the WINSTEPS Rasch computer software program version 3.91.0.0 [18]. The analyses were performed using a systematic stepwise approach similar to that used in previous studies [12, 19, 20].

First, an evaluation of the psychometric properties of the rating scale was conducted. The criterion used was that the average measures for each response category on each item should advance monotonically, as evidenced by an *Outfit Mean Square (MnSq)* value of less than 2.0 for each of the step calibrations [21].

The second step aimed to evaluate the fit of the item responses [17]. Any item that did not show acceptable goodness-of-fit to the Rasch model was removed, and the psychometric properties of the remaining items were re-analyzed until all items demonstrated acceptable goodness-of-fit, defined as *Infit MnSq* values between 0.7 and 1.3 logits [22]. In the third step, we evaluated the level of unidimensionality in the generated LFS measures through a principal component analysis (PCA) of the residuals, with the criterion that the first latent dimension should explain at least 50% of the total variance [23].

The fourth step evaluated aspects of person response validity. The criterion for evaluating person goodness-of-fit was to reject *Infit MnSq* values of 1.4 logits or higher or associated with a z-value of 2 or higher, accepting that 5% of the sample may by chance fail to demonstrate acceptable goodness-of-fit without threatening evidence of person response validity [24–26]. We also examined ceiling and floor effects by determining the number of respondents obtaining minimum and maximum scores on the scale. Up to 10% of the sample with minimum or maximum scores was considered acceptable.

In the fifth step, Differential Item Functioning (*DIF)* analyses were performed in order to evaluate the stability of the LFS response patterns in relation to diagnosis (stroke or osteoarthritis) using the Mantel-Haenzel statistics for polytomous scales using log-odds estimators [27, 28], as reported from the WINSTEPS program. Statistics with Bonferroni-corrected *p*-values < 0.01 were considered indicative of DIF.

The last two steps assessed several aspects of the scale’s reliability. In the sixth step, person separation reliability (i.e., ability to separate participants into distinct fatigue groups) was evaluated by calculating the scale’s person separation index [29]. The separation index reflects the number of statistically different groups that the scale can identify in the sample, considering the range and precision of individual person estimates. An index above 1.5 is required to ensure that the scale can differentiate people with at least two different levels of fatigue. Lastly, in the seventh step, we assessed the scale’s internal consistency reliability by reporting both Cronbach’s alpha reliability coefficient of the raw scores and Rasch-equivalent person reliability coefficient for the final unidimensional scale, as well as the Pearson correlation coefficient between the LFS sum scores and the Rasch-generated measures. Coefficients > 0.80 indicated acceptable internal consistency reliability.

In addition to the steps described above for psychometric analysis of the LFS, characteristics of the study samples were summarized and compared using SPSS statistical software, version 25 [30]. Differences in means and standard deviations (SD) between the two patient samples were assessed with independent sample t-tests for continuous and normally distributed variables. Categorical variables were assessed using frequencies and percentages, and the stroke and osteoarthritis samples were compared using Chi-square analysis. *P*-values < 0.05 were considered statistically significant.

## Results

Characteristics of the two patient samples are described in Table 1. Compared with the sample of stroke patients, the osteoarthritis sample had a larger proportion of women, had more education, and was more likely to be employed.

**Table 1:** Fatigue scores and demographic characteristics of the two patient samples and the overall sample

	Stroke sample (*n = 322*)	Osteoarthritis sample (*n = 203*)	*P*-value^1^	Total sample (*n = 525*)
Fatigue scores				
LFS (mean, SD)				
Item #1 *(N = 320; 200; 520)*	4.58 (2.33)	3.85 (2.45)	*0.001*	4.30 (2.40)
Item #4 *(N = 321; 200; 521)*	3.54 (2.30)	2.90 (2.17)	*0.002*	3.29 (2.27)
Item #5 *(N = 321; 201; 522)*	3.54 (2.32)	2.92 (2.14)	*0.002*	3.30 (2.27)
Item #16 *(N = 321; 201; 522)*	3.06 (2.38)	2.26 (1.95)	*< 0.001*	2.75 (2.25)
Item #17 *(N = 321; 200; 521)*	2.91 (2.56)	3.16 (2.62)	*0.286*	3.00 (2.27)
LFS-5 *(mean, SD) (N = 320; 198; 518)*	3.52 (1.89)	3.01 (1.92)	*0.003*	3.32 (1.92)
LFS-3 *(mean, SD) (N = 320; 199; 519)*	3.88 (2.10)	3.23 (2.13)	*0.001*	3.63 (2.14)
Demographics				
Age (years), *mean (SD)*	66.4 (12.8)	68.2 (9.2)	*0.061*	67.1 (11.6)
Sex, *n (%)*			*< 0.001*	
Male	190 (59.0)	64 (31.5)		254 (48.4)
Female	132 (41.0)	139 (68.5)		271 (51.6)
Level of education, *n (%)(N = 320; 198; 518)*			*< 0.001*	
Low (7–13)	217 (67.8)	96 (48.5)		313 (60.4)
High (14+ years)	103 (32.2)	102 (51.5)		205 (39.6)
Marital status, *n (%)(N = 322; 199; 521)*			*0.266*	
Married/partner	170 (52.8)	115 (57.8)		285 (54.7)
Not married	152 (47.2)	84 (42.2)		236 (45.3)
Cohabitation status, *n (%)(N = 322; 201; 523)*			*0.102*	
Live alone	104 (32.3)	79 (39.3)		183 (35.0)
Live with someone	218 (67.7)	122 (60.7)		340 (65.0)
Paid employment, *n (%)(N = 322; 200; 522)*			*< 0.001*	
No	310 (96.3)	130 (65.0)		440 (84.3)
Yes	12 (3.7)	70 (35.0)		82 (15.7)

^1^*P*-values of differences between the two samples

The LFS rating scale demonstrated acceptable outcomes in relation to the established criteria. In addition, all ten rating scale steps were used with a frequency above 100 scores for all scale steps. When analyzing the infit mean square statistics for the five included items, only one item out of five demonstrated acceptable goodness-of-fit (See Table 2). Two of the LFS items demonstrated higher than acceptable *MnSq* statistics (#16 *carry on a conversation* and #17 *close eyes*), and two items demonstrated lower than acceptable MnSq (#1 *tired* and #4 *fatigued*). Because items with higher fit statistics are a greater threat to unidimensionality compared to items with lower fit statistics, these two items were initially excluded and the remaining three items (#1, #4, and #5) were re-analysed. In the second iteration, the three remaining items demonstrated an acceptable range for goodness-of-fit to the model.

**Table 2:** Overview of the statistical approach, criteria, and results of the Rasch analysis of the LFS short form scale when used with people with stroke and osteoarthritis (*n* = 525)

**Aspect of validity evidence measured**	**Statistical approach and criteria**	**Initial LFS short form scale (5 items)**	**Final LFS short form scale (3 items)**
**Step 1:** **Evidence based on internal structure**	**Rating scale functioning** The average measures for each response category should advance monotonically, with outfit *MnSq* values < 2.0 for each of the step calibrations	Criteria were met	Criteria were met
**Step 2:** **Evidence based on internal structure (item misfit)**	**Item goodness-of-fit statistics** A sample-size adjusted criterion for item goodness-of-fit requiring infit *MnSq* values between 0.7 and 1.3	#17 close eyes: *MnSq =* 1.78 #16 conversation: *MnSq =* 1.42 #5 worn out: *MnSq* = 0.75 #1 tired: *MnSq =* 0.67 #4 fatigued: *MnSq =* 0.66	#5 worn out: *MnSq* = 1.02 #1 tired: *MnSq* = 1.10 #4 fatigued: *MnSq* = 0.80
**Step 3:** **Evidence based on internal structure (unidimensionality)**	**Principal component analysis of the residuals** The criterion was set for at least 50% of the total variance to be explained by the first latent variable	63.2%	81.6%
**Step 4:** **Evidence based on response processes**	**Person goodness-of-fit statistics** A criterion of < 5% of the sample has unacceptable person goodness-of-fit, as indicated by infit *MnSq* values < 1.4 and standardized z values < 2.0.	24 (4.6%)	29 (5.6%)
	**Floor and ceiling effects** A criterion of up to 10% of the sample could demonstrate minimum or maximum scores.	Maximum score = None Minimum score = 77 (14.0%)	Maximum score = 3 (0.6%) Minimum score = 88 (16.9%)
**Step 5:** **Item stability**	**Differential item functioning (DIF)** No Mantel-Haenzel statistics have a *p* < .01.	Items 4 and 5 demonstrated DIF by diagnosis	No item demonstrated DIF by diagnosis
**Step 6:** **Person-separation reliability**	**Person-separation index** An index of > 2.0 was required to ensure that the scale could differentiate the sample into at least three different levels of fatigue severity	Separation index = 1.82	Separation index = 2.49
**Step 7:** **Internal consistency**	**Person reliability (Rasch)** **Cronbach’s alpha coefficient (Cr-α**) **(raw scores)** **Relationship between individual sum scores and Rasch-based measures** The criterion was that all values should be > 0.80	Person reliability (Rasch) = .77 Cr-α (raw score) = .87 Correlation between sum scores and Rasch measures = .91	Person reliability (Rasch) = .89 Cr-α (raw score) = .91 Correlations between sum scores and Rasch measures = .98

*MnSq* Mean sq

The unidimensionality of both the 5-item and 3-item LFS scales was also acceptable, as the first latent variable accounted for 63.2 and 81.6% of the variance in the fatigue scores, respectively. Additionally, the proportion of the sample demonstrating misfit to the Rasch model for the 5-item LFS scale (4.6%) was within the set criterion of < 5% and was close to the criterion for the 3-item scale (5.6%). The number of respondents with maximum scores indicated negligible ceiling effects, but the number of respondents with minimum scores on both the 5-item and 3-item scales indicated a moderate floor effect.

The DIF analysis indicated that two of the misfitting items showed DIF in relation to disease group, but once those two items were removed, the 3-item scale revealed no systematic differences in relation to diagnosis across any of the three remaining LFS items. The separation index of the LFS scale also increased from 1.82 to 2.49 after deleting the two items demonstrating misfit, indicating the 3-item scale was able to distinguish three statistically distinct groups of fatigue. Measures of internal consistency indicated that the 3-item scale met all set criteria and performed better than the 5-item LFS scale. See Table 2 for a summary of the findings.

## Discussion

Findings from our study showed that a 3-item version of the LFS had better psychometric properties than the 5-item version. The 3-item LFS showed unidimensionality, accounted for a large proportion of explained variance, and was able to differentiate three statistically distinct fatigue severity groups. In a previous psychometric evaluation in a sample of women with HIV [14], all 13 items in the original LFS met the set criteria for item goodness-of-fit (internal validity) and explained 52.1% of the variance in scores. However, in studies of fatigue in which participants often experience a sense of exhaustion, lack of energy or tiredness distinct from sleepiness, shorter scales that are less burdensome for patients to complete would be preferable.

As shown in this analysis, scales with more items are not always better. They may lack unidimensionality, which poses challenges to the generation of meaningful total scores and may indicate that the use of subscales is warranted. Moreover, the inclusion of poorly performing items may actually reduce the ability of the scale to distinguish distinct groups based on level of severity, as occurred in this analysis, in which the 5-item scale could only distinguish two severity groups, while the 3-item scale was able to distinguish three severity groups. Even though these groups are based on statistical calculations, future studies could explore the clinical relevance and potential cut-offs for determining such group allocations. This could be a logical step for future research now that there is evidence that the 3-item scale is sensitive enough to detect statistically distinct groups.

Another interesting aspect is that the items in the 3-item version all demonstrated acceptable goodness-of-fit within the set ranges, indicating that the response patterns all contribute to the underlying measure, without evidence of over- or underfit as in the 5-item version, supporting validity evidence of internal structure. The three remaining items (Worn out; Tired; Fatigued) are conceptually more similar than the excluded items that involve interactions or behaviors (Conversation; Open eyes), also indicating evidence of validity of the test concept.

There were moderate floor effects for both the 3- and 5-item LFS, but the 3-item version had a higher mean value than the 5-item version. The low severity scores may have been due to our reduction of the rating scale from 0 to 10 to 1–10. However, other studies on the LFS have also reported floor effects [13], so this may be an issue with the LFS regardless of the slightly modified rating scale. Another likely explanation for the moderate floor effects is that many of the patients in these two samples were not experiencing fatigue or were limiting their activity to minimize their fatigue.

The 3-item LFS was not biased by diagnosis, as indicated by the lack of DIF and similar response patterns across two different patient groups. Combined with the results of prior studies among patients with cancer and HIV [13, 14], this study provides additional evidence that short versions of the LFS can be used as a generic PROM measure of fatigue. In particular, the finding that the items retained in this 3-item version of the LFS were also retained in a 5-item version of the scale evaluated among women living with HIV [2] suggests some degree of consistency, even across different patient populations and across different languages.

One challenge resulting from the use of different short versions of the LFS is how to compare scores from versions containing different items. One solution might be to use Rasch analysis to generate a stable and disease-generic item hierarchy that can be used to select subsets of items for specific studies and still generate comparable measures through conversion tables or computer-adaptive testing (CAT) procedures. Even though some similarities in the item hierarchies from our earlier studies occur in relation to these findings [14], more in-depth analyses with larger samples are required in order to establish such a disease-generic item pool.

Based on the findings from this study, the idea may arise that a single “perfect” fatigue item could perform as well as multi-item versions assessing subtly different aspects of fatigue. Although this could be explored in this sample and others where the LFS has been evaluated using Rasch analysis [13, 14], another body of evidence from qualitative research suggests that multiple items may be necessary, as the phenomenon of fatigue may be perceived by patients in multiple and complex ways [31–33]. Thus, the balancing act between developing psychometric excellence and measuring fatigue’s complex manifestations is likely to continue.

The translation of any PROM requires the use of stringent procedures to ensure conceptual equivalence in the new translation compared with the original language version [34]. Conceptual equivalence is closely linked to cultural relevance, as culture is a primary determinant of language [34]. A lack of conceptual equivalence and cultural relevance may lead to a risk of misinterpretation of items or concepts and, consequently, low content validity in the translated version. One challenge with the Norwegian language translation of the 5-item LFS used in this study is that the wording of each item is difficult to differentiate in the Norwegian language. In the stroke study, the LFS was administered as an in-person assessment interview, which helped in delineating the respondents’ understanding of each concept. Some respondents had difficulties in distinguishing items 1 and 5, and this was discussed during the assessment. However, our analysis shows that none of these items are redundant, as the goodness-of-fit indicates that both should be retained in the 3-item version of LFS.

### Strengths and limitations

The strengths of this secondary analysis included the relatively large samples from two diverse patient populations and the thorough evaluation of the psychometric properties. However, a significant limitation is that this study evaluated only five of the original 13 LFS items, so it remains unclear whether the three items retained in this analysis represent the best three items for inclusion in a brief fatigue severity PROM. It would therefore be interesting to determine whether the 3-item version generated in this analysis outperforms other potential short versions, particularly the 5-item short form developed in the prior study of women living with HIV [2].

In addition, this study evaluated a Norwegian version of the LFS. Translation of the concept of fatigue into Norwegian and perhaps other languages is difficult, since some English words and phrases do not have direct equivalents in Norwegian or other languages. Thus, generalization of the findings from this Norwegian study to other populations must be done with caution.

Finally, the mode of data collection was not identical in the stroke and osteoarthritis samples. The stroke sample was interviewed in person, while the osteoarthritis sample completed the questionnaire independently. Thus, the patients with stroke may have received help in understanding the meaning of the different items, whereas the patients with osteoarthritis were left to interpret the items on their own. Although no DIF was found based on diagnostic group, the differing data collection mode for the two samples may have introduced bias in the interpretation of the items and, thus, may have influenced the results.

## Conclusions

The results of this study indicate that a 3-item version of the Lee Fatigue Scale has acceptable psychometric properties and is sufficiently generic for use as a PROM for fatigue severity with patients post-stroke and patients living with osteoarthritis. Future research should be conducted to evaluate the validity of the 3-item version for use among other clinical populations.

## Data Availability

Datasets generated and analyzed during the current study are not publicly available due to strict ethics regulations in Norway but may be available from the corresponding author on reasonable request.

## Abbreviations

CAT: Computer-adaptive testing
DIF: Differential Item Functioning
HIV: Human immunodeficiency virus
IRT: Item response theory
LFS: Lee Fatigue Scale
MnSq: Mean Square
PCA: Principal Components Analysis
PROM: Patient-reported outcome measure
SD: Standard deviation
